# Polyaniline Based Electrochemical Sensor for the Detection of Dengue Virus Infection

**Published:** 2020

**Authors:** Reshmi Dutta, K Thangapandi, Sumantra Mondal, Amalesh Nanda, Shreyosi Bose, Shairee Sanyal, Saikat Kumar Jana, Suvankar Ghorai

**Affiliations:** 1. Department of Biotechnology, Faculty of Bioengineering, SRM Institute of Science and Technology, Kattankulathur, Tamil Nadu, India; 2. Department of Biotechnology, National Institute of Technology, Yupia, Papum Pare, Arunachal Pradesh, India

**Keywords:** Dengue virus, Dielectric spectroscopy, Electrodes, Polyaniline, Voltammetry

## Abstract

**Background:**

Dengue burden is increasing day-by-day globally. A rapid, sensitive, cost-effective early diagnosis kit is the need of the hour. In this study, a label-free electrochemical immunosensor was proposed for dengue virus detection. A modified Polyaniline (PANI) coated Glassy Carbon (GC) electrode, immobilized with DENV NS1 antibody was used to detect the circulating DENV NS1 antigen in both spiked and infected sample.

**Methods:**

Cloning, purification and expression of DENV NS1 protein in *Escherichia coli (E. coli)* was performed and sensor design, PANI modification on GC electrode surface by electrochemical polymerization and immobilization of NS1 antibody on the modified electrode surface was done and finally the analytical performance of the electrochemical immunosensor was done using Cyclic Voltammetry (CV) and Electrochemical Impedance Spectroscopy (EIS).

**Results:**

CV and EIS were used to study and quantitate the circulating DENV antigen. The calibration curve showed wide linearity, good sensitivity (Slope=13.8% IpR/*ml.ng*^ −1^) and distribution of data with a correlation coefficient (R) of 0.997. A lower Limit of Detection (LOD) was found to be 0.33 *ng.ml*^ −1^ which encourages the applicability of the sensor.

**Conclusion:**

Thus, a PANI based new electrochemical immunosensor has been developed which has the potential to be further modified for the development of cost effective, point of care dengue diagnostic kit.

## Introduction

*Aedes albopictus* and *Aedes aegypti* are the carriers of dengue virus, responsible for dengue infection. The DENV genome is 11 *kb* in length and has a single positive stranded RNA. There are four antigenically distinct serotypes DENV1, DENV2, DENV3, and DENV4 1.

About 2.5 billion people worldwide are currently affected by dengue virus, with an annual surplus of 50–100 million infections mostly affecting children 2. Increasing geographic expansion to new countries has led to an upsurge of about 30-fold in incidents in the last 50 years. Recent estimates make it clear that there are 390 million cases of dengue per year, of which 96 million are manifested clinically. From another study across 128 countries, it is reported that 3.9 billion people were affected by dengue virus 2. In India, there are recent reports of DENV-2 infected patients as compared to other serotypes which clearly indicates the sero- dominance of DENV-2 3–5.

In order to have a better treatment alternative, precise and methodological detection of dengue is very crucial. After the onset of symptoms, serum, plasma, circulating blood cells and other tissues can contain the virus for 4–5 days 6. Virus isolation, nucleic acid and antigen detection are some of the methods to diagnose the disease in the early stages 7. Serology is used for diagnosis at the end of acute infection phase. Reverse Transcription Polymerase Chain Reaction (RT-PCR), though specific, is a costly, laborious and time consuming method 8. Serological tests like Hemagglutination Inhibition (HI) assay and Enzyme Linked Immunosorbent Assay (ELISA) for detection of dengue infections are relatively cost effective and easy to handle, but time consuming and require experts to operate 9. Moreover, some hidden challenges like lack of specificity and sensitivity of the identified biomarkers pose a problem for researchers 7, 10.

Biosensors are beneficial as compared to other traditional techniques because they are precise, cost-effective, quick and simultaneous detection of multiple analytes can be performed easily 11. Electrochemical immunosensors are favorable for the detection of multiple analytes, are unaffected by the volume of the sample and can be used for colored complex samples 12. Electrochemical sensors can be divided into two groups- labelled and label-free 13. In labelled biosensors, the electrochemical response is dependent on active redox markers and changes its concentration upon interaction of the analyte with immobilized substrate 14. The redox markers for labelled electrochemical immunosensors are mostly dyes or enzymes which interact with the analytes or have binding selectivity 15–17. Label-free electrochemical immunosensors are advantageous since they reduce the steps for detection. Direct detection eradicates the marking steps, reduces the time and is cost-effective 10, 18–20. Though biosensors can work as a substitute for traditional and conventional tests for detection of dengue virus, they have some shortcomings which avert their commercialization for detection of dengue infection like lack of sensitivity, reusability, false positive results and need for tagging 21. The most commonly used techniques for label-free electrochemical detection are Cyclic Voltammetry (CV) and Electrochemical Impedance Spectroscopy (EIS) 22. The interest in CV and EIS can also be attributed to the possibility of collecting spectra over a wide range of frequencies, allowing the complete characterization of the surface in a short time interval 23, 24.

Cavalcanti *et al* reported label-free immunosensor for dengue virus infection using gold electrode which achieved the Limit of Detection (LOD) of 0.33 *ng.ml*^−1^ 10. In another study, an impedimetric immunosensor was used for testing neat serum for dengue diagnosis as reported by Juliana Cecchetto and the LOD was 3 *ng.ml* ^−1^ 25. Gold electrodes can easily accomplish self-assembly *via* gold–thiol interactions, but their high electrochemical active surface is easily inactivated by modification, and their potential window is limited to a relatively positive range due to low over potential of gold for hydrogen evolution 26. Mian Hasnain Nawaz *et al* 27 reported development of a portable and disposable NS1 based electrochemical immunosensor for early diagnosis of dengue virus. Electrochemical detection of dengue virus NS1 protein was done using poly (Allylamine)/carbon nanotube layered immunoelectrode by Mízia M. S. Silva 28 and the obtained LOD was 0.035 *μg*/ml^−1^ which is not considerable for clinical measurement. Helena P.O. Nascimento *et al* 29 proposed an impedimetric biosensor using sensitive layer based on gold nanoparticles-polyaniline hybrid composite (AuNpPANI) for detection and monitoring the hybridization of dengue viruses serotype at picomolar concentration. This approach, although effective, may increase the risk of non-specific reactions in dengue immunoassays since various antibodies are produced for different viral proteins. Figueiredo *et al* reported electrical detection of dengue biomarker using egg yolk immunoglobulin with LOD of 0.09 *μg.ml*^−1^ which was not suitable for clinical application 30.

According to the current studies, one of the well-known early biomarkers for DENV virus is non-structural protein NS1, released into the blood stream in moderately high concentrations during replication 31, 16. Many electrochemical immunosensors have been developed for early detection of NS1 protein in patient serum within a wide detection range and low LOD 8.

In our present study, an electrochemical immunosensor was developed based on glassy carbon (GC)/Polyaniline (PANI) which has very a wide detection range and low LOD and is a cost effective method for point of care diagnosis of NS1 antigen from affected serum. Polyaniline (PANI) is a conducting polymer and has been used as a transducer material for biosensing applications. This work shows the potential use of PANI in electrochemical immunosensors for label free detection of dengue virus 32.

## Materials and Methods

### Chemicals

Abcam products include NS1 protein (ab96036), and anti-NS1 mouse monoclonal antibody (ab47702). Sigma-Aldrich products include hydrochloric acid (HCl) (37%), sulphuric acid (H_2_SO_4_) (96%), sodium chloride (NaCl), glycine, potassium hexacyanoferrate (III) [(K_3_Fe(CN)_6_)], potassium hexacyanoferrate (II) trihydrate [(K_4_Fe(CN)_6_.3H_2_O)], potassium hydrogen phosphate (K_2_HPO_4_), potassium dihydrogen phosphate (KH_2_PO_4_), glutaraldehyde, aniline and Bovine Serum Albumin (BSA). Merck Millipore products include ultrapure water.

### Dengue virus type 2 NS1 cloning

Complementary DNA (cDNA) was used as template for entire genome PCR amplification. 5′ATGTGGA AGCAGATCACC 3′ with BamHI restriction site was used as a sense primer and 5′ GGCGGTCACCAGG CTGTTC 3′ with HindIII restriction site was used as an anti-sense primer. Following was the thermocycling conditions for 30 cycles: an initial denaturation for 5 *min* at 94°*C*, annealing at 94°*C* for 30 *s*, initial extension for 1 *min* at 55°*C* and 72°*C* for 1 *min*, with a final step of extension for 4 *min* at 72°*C*. After amplification, around 900 *bp* of DENV2 NS1 gene sequence flanked with BamHI and HindIII restriction sites were obtained. Qiagen gel extraction kit was used to purify the PCR products, followed by digestion with BamHI and HindIII restriction enzymes and then ligated into respective BamHI and HindIII restriction sites of the linearized pET21a (+) expression vector for generation of pET-NS1 recombinant plasmid. It was then transformed into competent *Escherichia coli* (*E. coli)* BL-21 cells. Selection of recombinant bacteria was done on LB agar containing ampicillin.

### Expression and purification of the rNS1 protein

The recombinant protein was expressed by inducing the pET system into *E. coli* host strain BL-21 in 300 *ml* LB broth containing 50 *μg.ml*^−1^ ampicillin. 0.5 *mM* isopropyl thio-galactoside (IPTG) was added for induction. Bacterial cultures were then grown for 3–4 *hr* and *E. coli* cells were collected by centrifugation followed with 3000x*g* at 4°*C* for 30 *min*. The cells were lysed by sonication and soluble protein fractions were obtained by centrifugation of the cell lysate at 20,000x*g* for 20 *min* at 4*°C*. Affinity chromatography for purifying NS1 6X His-tagged protein was performed using 3 *ml* Ni^2+^ nitrilotriacetic acid (Ni-NTA) resin. Protein fractions were washed and eluted with 250 *mM* imidazole in buffer A. Protein was estimated by Bradford assay. SDS-PAGE followed by Coomassie blue staining and western blotting was performed to determine the expression level and purity of protein. The Western Blot procedure has been followed according to the standard protocol.

### Sensor design and surface modification

Glassy Carbon Electrode (GCE) was mechanically actuated by polishing with alumina slurry (0.3 *μm* and 0.05 *μm*) to get specular-like surface and was thoroughly sluiced with double distilled water. GCE was sonicated for 5 *min* in double distilled water and absolute ethanol was used for cleaning. After drying, GCE was electrochemically activated using 0.1 *M* H_2_SO_4_ solution, followed by cyclic voltammetry in an electrochemical workstation. The potential range was set from −0.2 *V* to +1.6 *V* at a scan rate of 100 *mV*.s^−1^. 0.05 *M* aniline solution was prepared in 0.1 *M* PBS. Aniline was deposited and polymerized on the surface of GCE by applying potential between 0.0 and +0.1 *V* at a scan rate of 100 *mV.s*^−1^ for 10 cycles using cyclic voltammetry. Polyaniline (PANI) modified GCE electrode was immediately immersed in 1% glutaraldehyde solution for 15 *min* before immobilization of antibodies on the modified PANI-GCE surface.

### Antibody immobilization

Anti-NS1 antibody in 0.1 *M* PBS (pH=7.4) was applied over the electrode and incubated at 4°*C* for 12 *hr*. Nonspecific binding sites of the working electrode were blocked with 100 *μl* of 0.01 *mg.ml*^−1^ BSA in 0.1 *M* PBS (pH=7.0) for 30 *min* at 4°*C*. The electrode was then washed with 0.1 *M* PBS buffer (pH=7.0) and stored at 4°*C*.

### Electrochemical measurements

Glassy Carbon (GC), silver (Ag/AgCl) and Platinum (Pt) wires were used as working, reference and counter electrode, respectively. 10 *mM Fe*(CN)_6_^3−/4−^ was used as electroactive indicator with 0.1 *M* PBS solution (pH=7.4) to perform cyclic voltammetry. Square Wave Voltammetry (SWV) was set at 15 *Hz*, scan increment at 4 *mV* and amplitude at 25 *mV*. Electrochemical measurements were executed employing electrochemical workstation Biologic SAS (SP-150).

### NS1 electrochemical detection

1 *μg*.*ml*^−1^ of NS1 recombinant purified protein in PBS was prepared. The dengue infected serum samples with different dilutions were also prepared for detection. Both of these samples were placed over the immunosensor electrode and electrochemical measurements were performed.

### Regeneration of NS1 immunosensor

**Figure 1.**
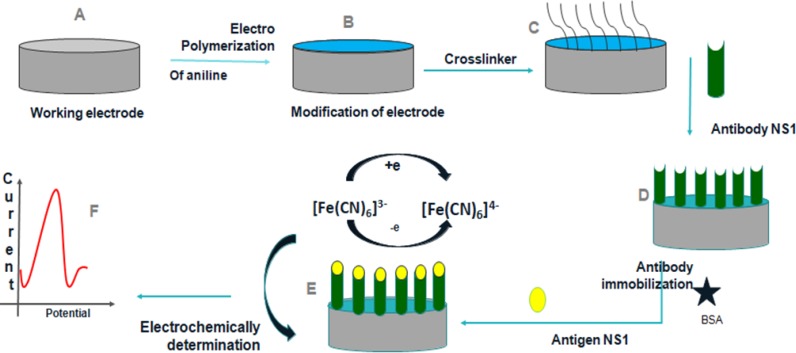
Schematic representation of construction and development of the immunosensor.

In order to regenerate the sensor for further use, few steps were performed. After NS1 detection, the immunosensor electrode was submerged in glycine-HCl solution (0.1 *M*) at pH=3 at 4°*C* for 20 *min*. Later, sensor was rinsed thrice with PBS for further NS1 determination (Figure 1).

### DENV 2 NS1 ELISA

ELISA was performed to optimize the concentration of dengue virus NS1 antigen and antibody. The purified antigen (Also commercial NS1 antigen) was diluted to 20 *μg.ml*^−1^ with carbonate buffer (0.1 *M* NaHCO_3_, pH=9.2). The experimental wells of the 96-well ELISA plate (Nunc-Immuno plate, Polysorp) were coated with 50 *μl* of NS1 antigen. A saran wrap was then used to wrap it so that the antigens get bound to the wells and was kept for 2 *hr* at 37*°C*. After incubation to remove any unbound antigens from the wells, 1×PBS having 0.1% Tween-20 was used to rinse the wells thrice. To block any unoccupied sites, all the wells were filled up with 250 *μl* blocking buffer (1% casein in 1×PBS) and kept for 1-*hr* incubation at room temperature. After discarding the blocking buffer, the wells were washed thrice. 10^−1^, 10^−2^, 10^−3^, 10^−4^, 10^−5^ and 10^−6^ dilutions of the NS1 antibody were prepared and 50 *μl* of the dilutions were introduced over the antigen coated wells. Following incubation at room temperature for 1 *hr*, plates were washed with 1×PBS containing 0.1% Tween-20 to remove unbound primary antibody. Horseradish Peroxidase (HRP) enzyme conjugate (Amersham) being the secondary reagent, was diluted 200 times in a solution of 1× PBS and 0.5% BSA. 50 *μl* of this conjugate was added to all wells and incubated for 1 *hr* at room temperature. PBS containing 0.1% Tween-20 was used to wash the wells thrice to remove any trace of unbound protein A conjugate. 0.1 *M* citrate buffer (pH=−4.0) was used to prepare ABTS [2,2′-Azino-bis-(3-ethylbenzthiazoline-6-sulphonic acid)] substrate solution at 1 *mg.ml*^−1^ concentration. 50 *μl* of this solution with 0.2% H_2_O_2_ was added to all the wells and kept for 5 *min* at room temperature for color development. The O.D. was measured at 405 *nm* wavelength. The detection of different NS1 dilutions, 10^−1^, 10^−2^, 10^−3^, 10^−4^, 10^−5^ and 10^−6^, by ELISA was used as a reference for the initial sample range added to the electrochemical immunosensor.

## Results

### Cloning, expression and purification of DENV NS1 protein in *E. coli*

In order to amplify the ORF of DENV2 NS1, two primers were used: forward primer (5′ GGATCCATG TGGAAGCAGATCACC 3′) and reverse primer (5′ A AGCTTGGCGGTCACCAGGCTGTTC 3′) were designed and synthesized. PCR amplification has been done, followed by agarose gel analysis. An amplified PCR product of 858 *bp* was excised from the gel and subjected to restriction enzyme digestion. Insert was ligated with digested vector (pET-21a) followed by transformation in *E. coli* cells. Protein was induced by IPTG and purified by Ni-NTA chromatography. Finally, the single eluted fractions were concentrated by Amicon filter and the concentration was determined by Bradford assay.

**Figure 2.**
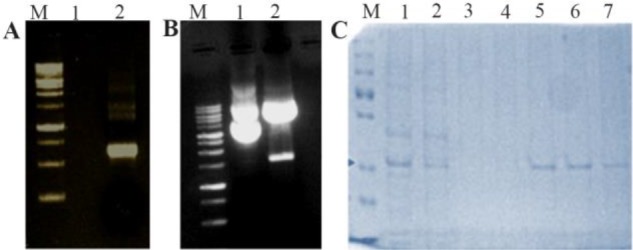
Expression and purification of DENV NS1 in bacteria. A) PCR amplified product (Lane M: Molecular weight marker, Lane 1: Negative control, Lane 2: PCR amplified NS1 product). B) Cloning of DENV NS1 gene in pET-21a vector (Lane M: Molecular weight marker, Lane 1: Undigested cloned plasmid Lane 2: Digested cloned plasmid). C) SDS-PAGE analysis of DENV NS1 protein (Lane M: Protein molecular weight marker, Lane 1: Expressed crude cell lysate, Lane 2: Flow through, Lane 3–4: Wash, Lane 5–8: Eluted protein). The concentrated recombinant protein was used for the calibration of the designed sensor along with the original patient sample.

The amplified NS1 gene having 858 *bp* was clearly seen in the 1% agarose gel as shown in figure 2A. The pET-21a vector and insert NS1 DNA were digested with BamHI and HindIII as shown in figure 2B and insert NS1 DNA was cloned into pET-21a vector to produce the recombinant pET-21a-NS1.

After successful cloning of NS1 in pET-21a, the positive clone was transformed to *E. coli* BL-21 strain competent cells and plated in LB-ampicillin plate. The positive clone was induced by 0.5 *mM* IPTG. The NS1 protein was purified by Ni-NTA chromatography followed by 15% SDS-PAGE analysis. As shown in figure 2C, a purified band of around 25 *kDa* was observed. Finally, the single eluted bands were pooled and filtered through Amicon filter (Millipore). The final yield was determined by Bradford assay and found to be 8 *mg.ml*^−1^.

### Morphology of polyaniline

**Figure 3.**
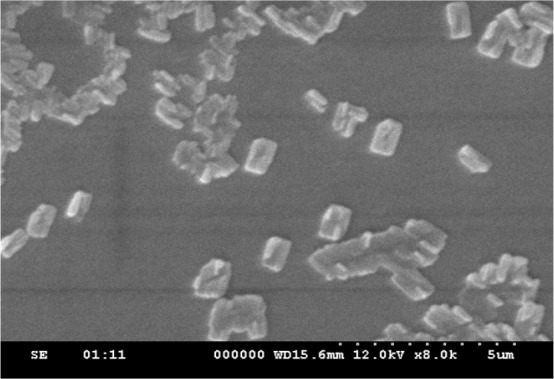
SEM image of Polyaniline.

The Scanning Electron Microscope (SEM) image represents the “rectangular” shaped PANI deposited on the surface of GC with dimension of approximately 0.5×1 *μm* (Figure 3).

### Electrochemical polymerization

**Figure 4.**
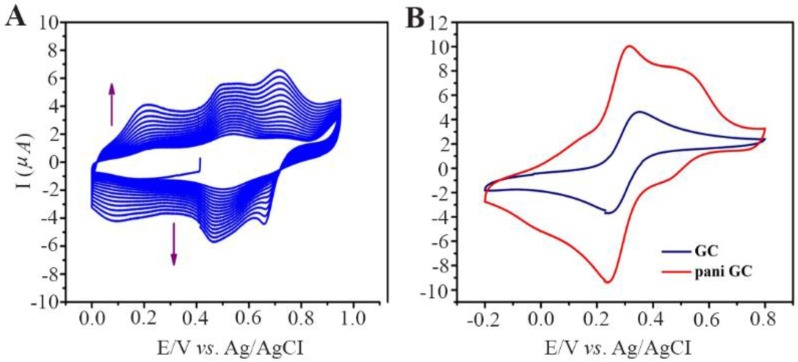
A) Electrochemical polymerization of polyaniline on Glassy carbon, cyclic voltammetry in 0.05 *M* aniline 0.1 *M* PBS pH=3.5 at 100 *mV/s*. B) Evidence of PANI modified GC and bare GC.

GC/PANI electrode was surface modified by electrochemical polymerization. The protocol was followed according to the redox processes of electron transfer over the electro-deposited PANI film. Similar trends were observed in both experiments which are presented in figure 4 (A and B). Figure 4A represents polymerization of aniline which is confirmed in figure 4B where GC/PANI showed increased conductivity. Multicycle CV result clearly displayed an increase in redox peak current indicating the deposition of polyaniline on the surface of the electrode. Figure 4 shows multipeaks which can be a result of over oxidation of polyaniline.

### Electrochemical behavior of modified electrodes

**Figure 5.**
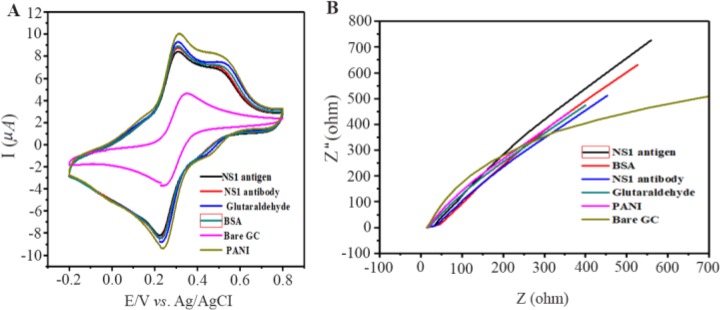
Behaviour of PANI modified GCE electrode (A) Cyclic voltammograms. (B) Impedance spectroscopy of different modified electrodes using 0.1 *M* PBS pH=7.6 containing 10 *mM Fe*(CN)_6_^3−/4−^.

Characteristics of modified electrodes can be studied by Cyclic Voltammograms (CV). CV measurements were taken in 0.1 *M* PBS (pH=7.6) containing 10 *mM* Fe(CN)_6_^3−/4−^. The scanning rate was set at 20 *mV*^−1^ and ranged from −0.2 to 0.6 *V*. Figure 5A depicts conductivity of electrode that was increased when bare GC electrode was modified with PANI. Later, there was a constant decrease in conductivity in the subsequent stages of antibody immobilization and antigen binding. Electrochemical Impedance Spectroscopy (EIS) was then performed to monitor the change in interface property of the electrode surface (Figure 5B). At the same environment of CV, the readings of EIS show that bare GC electrode has highest impedance and it gets reduced to a great extent when modified with PANI thus supporting CV measurements. EIS showed a trend of increase in impedance when PANI modified electrodes are coated with antibody and exposed to antigen solution.

### A nalytical performance of the electrochemical immunosensor

**Figure 6.**
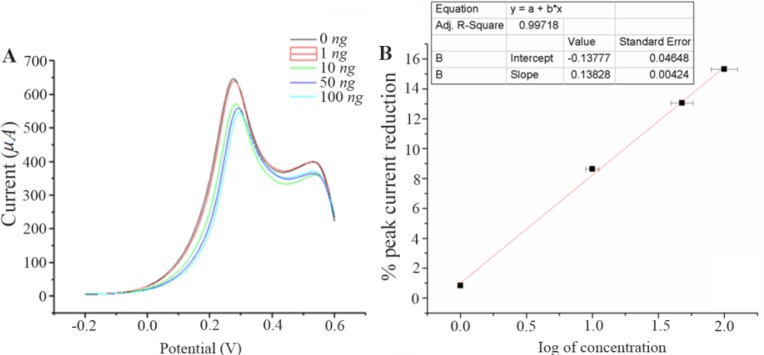
A) Square wave voltammograms of different concentration of NS1 in 0.1 *M* PBS pH=7.4 containing 10 *mM Fe*(CN)_6_^3−/4−^. B) Calibration plot for the immunosensor based on the percentage of SWV cathodic peak current reduction against the NS1 concentration using 10 *mM*.

The developed immunosensor was used to detect in-house expressed NS1, commercially purchased NS1, samples spiked with different concentrations of NS1 as well as NS1-positive blood samples. In all the cases, the immunosensor detected a decline in peak current as the antigen concentration increases (Figure 6A). This trend is supported by the fact that more binding of NS1 antigen to the antibody decreases electron flow rate at the electrode surface.

The graph between %I_p_R and log of NS1 antigen concentration is found to be a straight line and can be portrayed as the calibration curve for unknown NS1 concentration. The linearity is established at low concentration range of 1 and 100 *ng.ml*^−1^ (Figure 6B). It shows an appreciable reproducibility and a LOD of 0.33 *ng.ml*^−1^ which can be found useful clinically (LOD <1). Apart from having a wide range of linearity and low LOD value, this technique of label free NS1 detection by the fabricated immunosensor exhibited several other advantages like avoiding the use of enzyme-linked antibody thus reducing the cost of manufacturing. The response of the proposed sensor was compared with the previously reported immunosensors by various researchers and is represented in table 1.

**Table 1.** Summary of comparative study of various types of biosensors

**Sl. No.**	**Material and electrode used in biosensor**	**Detection technique**	**Linear range (*ng.ml*^−1^)**	**Limit of detection (*ng.ml*^−1^)**	**Reference**
**1**	Potassium ferricyanide and gold film electrode	DVP	1–100	0.33	Igor T. Cavalcanti (2015)
**2**	Gold nanoparticles and screen printed gold electrode (SPGE)	CV	1–25	0.5	Varun Rai (2016)
**3**	Mercaptoundecanoic acid, and gold electrode	EIS, CV	10–1000	30	Juliana Cecchetto (2015)
**4**	Egg yolk immunoglobulin (IgY) and Au electrode	CV	100–10000	90	Juliana F. dos Santos (2015)
**5**	Bovine serum albumin(BSA) and modified screen printed carbon electrodes (SPCE)	DPV	1–200	0.3	Mian Hasnain Nawaz (2018)
**6**	Horseradish peroxidase enzyme (HRP) and screen printed carbon electrodes (SPCEs)	CV	500–2000	30	Om Prakash (2014)
**7**	Dimethylformamide (DMF) and poly (Allylamine) carbon nanotube layered immunoelectrode	CV	10–50	0.49	Mízia M. S. Silva (2014)
**8**	Polyaniline (PANI) and glassy carbon electrode (GC)	CV	1–100	0.1	This Work

### Re-generation, reproducibility, stability and specificity of the immunosensor

**Figure 7.**
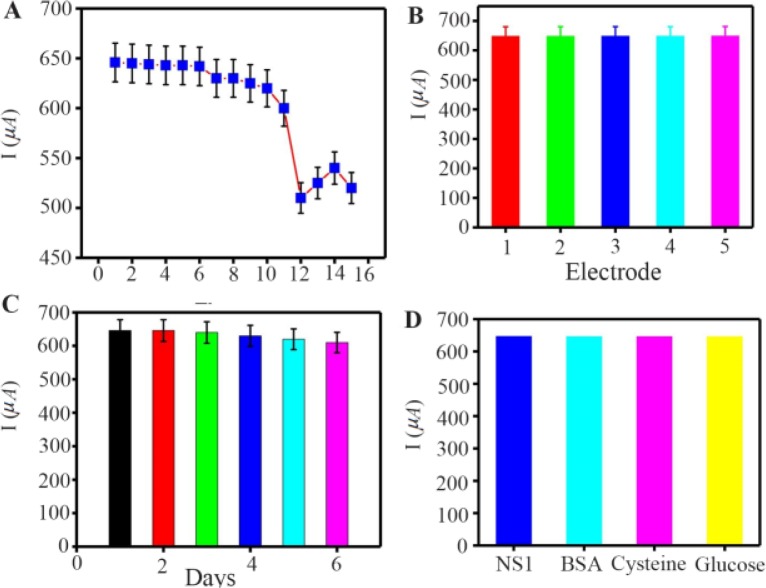
A) Current response regeneration of fabricated immunosensor, B) reproducibility, C) storage stability. D) current response selectivity of the immunosensor in the presence of 1 *ng.ml*^−1^ NS1, 1 *ng.ml*^−1^ NS1 + 10 *ng.ml*^−1^ BSA, 1 *ng.ml*^−1^ NS1 + 10 *ng.ml*^−1^ Cysteine, 1 *ng.ml*^−1^ NS1+ 10 *ng.ml*^−1^ Glucose SWV response of corresponding. Error bar = RSD (n = 3).

Evaluation of regeneration, reproducibility, stability and specificity is significant to prepare robust immunosensors for clinical use. The reusability, reproducibility, stability and specificity of the immunosensor were evaluated by SWV detection of 1 *ng.ml*^−1^ samples of NS 1 antigen in PBS (pH=7.4). The proposed immunosensor was examined for six cycles of regeneration and 11% of the initial signal was recovered (Figure 7A). Reproducibility refers to the variation in the average measurements of different sensors following a particular protocol after optimization. Reproducibility of our fabricated sensor is shown in figure 7B, where five different immunosensors are used for NS1 antigen detection (1 *ng.ml*^−1^) in 1 *ml* PBS (pH=7.4). The Relative Standard Deviation (RSD) of five repeated measurements was 1.9% indicating practical application of the designed immunosensor.

The stability of the immunosensor was studied and shown in figure 7C. After incubation with 1 *ng.ml*^−1^ NS1, the successive SWV scans for 60 cycles were recorded and the current change was less than 5%, representing satisfactory stability of the proposed immunosensor. Moreover, long-term stability of the biosensor was examined for a time of 15 days. In the case of storing the biosensors at 4*°C* (in dry form), the current still remained 89.7% after 6 days, which shows that the response was acceptable and the proposed immunosensor had good stability.

## Discussion

To evaluate the specificity, BSA, cysteine, and glucose contained in human serum were used as the interfering substances to test the selectivity of immunosensors. The current response in the presence of interfering substances, including NS1 (1 *ng.ml*^–1^) and BSA (10 *ng.ml*^–1^+1 *ng* NS1), cysteine (10 *ng.* *ml*^–1^+1 *ng* NS1), glucose (10 *ng.ml*^–1^+1 *ng* NS1) were measured by SWV. Figure 7D shows clearly that the current variation does not exhibit any significant influence (less than 7%) due to the presence of interfering substances.

### Human serum samples analysis

For the early detection of dengue, serum NS1 level determination is important. Serum samples of human were prepared with known amount of NS1 and validation of the method was performed. The accuracy of this immunosensor method was determined with comparison from the ELISA dengue analysis kit (Abcam) as shown in table 2.

**Table 2.** Blood sample analysis

**Source**	**Proposed sensor**			**Reference method ELISA**	

	**Added (*ng.ml*^−1^)**	**Found (*ng.ml*^−1^)**	**Std. Dev (n=3)**	**Found (*ng.ml*^−1^)**	**Std. Dev (n=3)**
**Spiked blood serum samples**	5	4.5, 4.2, 4.7	0.251	4.6, 4.5,4.7	0.1
	10	9, 8.9, 9.3	0.189	9.2, 9.3, 8.5	0.43
	30	25, 27,23	2	27,28, 27.8	0.52

## Conclusion

In this work, an approach was described for detection of dengue NS1 protein *via* label-free electrochemical immunosensor based on PANI-modified GC electrode. ELISA dengue analysis kit was used to compare the results obtained from the immunosensor and examine the accuracy of NS1 determination. The immunosensor showed detection for in-house expressed, commercially purchased, spiked and blood NS1 samples, which confirmed its sensitivity over a broad range of samples. The calibration curve showed broad range linearity and good sensitivity, where the slope was determined to be 13.8% I_p_R /*ml.ng*^−1^. A good distribution of the data with a correlation coefficient (r) of 0.997 was found. LOD was found to be 0.33 *ng.ml*^−1^. The experiment was performed in triplicate and has shown good reproducibility. The fabrication of these devices is economical and proves to be an effective alternative to conventional ELISA testing. Moreover, merging of point-of-care diagnosis and treatment of dengue can be possible.
